# Dynamic stability and spatiotemporal parameters during turning in healthy young adults

**DOI:** 10.1186/s12938-018-0558-5

**Published:** 2018-09-21

**Authors:** Chuan He, Rui Xu, Meidan Zhao, Yongming Guo, Shenglong Jiang, Feng He, Dong Ming

**Affiliations:** 1 Lab of Neural Engineering & Rehabilitation, Department of Biomedical Engineering, College of Precision Instruments and Optoelectronics Engineering, Tianjin University, Tianjin, China; 2 Tianjin International Joint Research Center for Neural Engineering, Academy of Medical Engineering and Translational Medicine, Tianjin University, Tianjin, China; 3 College of Acupuncture and Massage, Tianjin University of Traditional Chinese Medicine, Tianjin, China

**Keywords:** Gait analysis, Turning, Dynamic stability, Step turn, Spin turn, Margin of stability, Gait stability, Spatiotemporal parameters

## Abstract

**Background and purpose:**

Turning while walking has a frequent occurrence in daily life. Evaluation of its dynamic stability will facilitate fall prevention and rehabilitation scheme. This knowledge is so limited that we set it as the first aim of this study. Another aim was to investigate spatiotemporal parameters during turning.

**Methods:**

Fifteen healthy young adults were instructed to perform straight walking, 45° step turn to the left and 45° spin turn to the right at natural speed. Dynamic stability was measured by margin of stability (MoS) in anterior, posterior, left and right direction at each data point where significant differences were detected using 95% bootstrap confidence band. Common spatiotemporal parameters were computed in each condition subdivided into approach, turn and depart phases.

**Results:**

Results showed that minimum anterior MoS appeared at middle of swing while minimum lateral MoS at contralateral heel strike in all conditions. Posterior MoS decreased before middle of turn phase in spin whereas after middle of turn phase in step. Lateral MoS and stride width declined in turn phase of spin while in depart of step. Spin had a long step and stride length. Long swing phases were observed in turns.

**Conclusions:**

These data help explain that people are most likely to fall forward at middle of swing and to fall toward the back and the support side at heel strike. Our findings demonstrate that instability mainly exist in turn phase of spin and depart phase of step turn.

**Electronic supplementary material:**

The online version of this article (10.1186/s12938-018-0558-5) contains supplementary material, which is available to authorized users.

## Background

Turning during ambulation is crucial to daily life. Turning steps constitute a considerable percentage (about 20–50%) of steps taken during activities of daily living [1, 2]. Turning increases the risk of falling caused by slipping compared with that caused by slipping while straight walking [3]. Falling while turning causes a 7.9 times increase in hip fractures than falling during straight walking [4]. Giving insight into turning gait may contribute to the evaluation and the development of rehabilitation program for movement disorders, design of assistive devices, gait planning of biped robot and animation design in computer animation industry.

According to whether there are obvious transition steps or not, turns can be divided into “steady-state” turns, such as circular walking, and “transient” turns, such as 90° turns [5, 6]. The latter can be performed using two turning strategies: (1) step turn, turning toward the contralateral side of the stance limb (outside leg strategy); (2) spin turn, turning to the ipsilateral direction of the stance limb (inside leg strategy) [7].

Researches show that there exists turning preference. Dixon et al. [8, 9] suggested that both typically developing children (n = 54) and cerebral palsy children (n = 22) preferred spin turns, while step turns were singly performed by only 1/54 and none respectively. Patla et al. [10] reported that healthy young adults performed step turns during more than 80% of trials when executing 60° turns while walking. Akram et al. [11] found that healthy older adults (n = 19, age = 66 ± 4.2 years) preferred spin turns, whereas step turns were preferred only in 90° turns while walking fast. But Conradsson et al. [12] revealed that step and spin turns followed a nearly 50:50 distribution during 90° turns both for 19 individuals with Parkinson’s disease (age = 72 ± 4 years) and for 17 age and gender matched healthy controls. Now, the reason for the turning preference is still unknown, although some studies [8, 12–14] have investigated the kinematics and kinetics during turning in people of different age groups. It is all-important to understand the intrinsic difference between step and spin turns.

Because turning increases risk of falling [3] and injury [4], we focused on the gait stability of turns in this study. We found that the stability evaluation of turning was limited, even though the assessment method of gait stability had been greatly developed [15, 16]. Stability is the capacity to maintain balance during perturbations [17]. Static stability, which is generally defined as ‘center of mass (CoM) should be vertically projected within base of support (BoS)’ [18], is widely accepted to assess stability of posture. Gait stability is usually evaluated by dynamic stability because gait is a dynamic movement. Dynamic stability can be divided into local dynamic stability and global dynamic stability depending on small and large perturbations respectively from internal and external sources [15, 16, 19]. Bruijn et al. [15] assessed four levels of validity of common gait stability measures and found that measures with high validity included largest Lyapunov exponent and variability measures in local dynamic stability, and foot placement estimator and margin of stability in global dynamic stability. Local dynamic stability, derived from dynamical systems theory, requires collecting kinematic data in a large number of continuous strides (usually around 110 strides [20]), which essentially determines that the measures derived from dynamical systems theory might not be practical for assessing the dynamic stability of a transient turn because it is only a small part of the walking trajectory. Global dynamic stability describes the probability of falling [21]. Foot placement estimator is high validity but in need of a full body marker set [15]. Margin of stability (MoS), proposed by Hof et al. [18], is extensively used due to high validity [15] and simple operation (requiring a few continuous gait cycle samples and no force plate). Based on the inverted pendulum model of balance, MoS can be used to assess gait stability for straight walking, for starting and stopping walking and for turning [22], and is much more convincing than the static method by considering both the relative position and velocity between CoM and BoS [18]. However, most studies investigating turning gait stability either assessed static stability, such as distance between the ankles [13, 23], stride width [12, 24] and distance between CoM and BoS [9, 13, 23, 24], or did not compare dynamic stability between two turning strategies and straight walking [9, 23]. For example, spin turns are generally considered less stable and demanding a higher biomechanical cost than step turns, mainly because Taylor et al. [13] indicated that the spin turn had a narrow BoS leading to CoM outside displacement and required increased joint moments and power, compared to the step turn and straight gait.

Furthermore, both turning strategies demand modification of spatiotemporal parameters from those of straight walking, which can somewhat reflect biomechanical adaptations simply and effectively [8]. Previous study [25] revealed that young adults decreased velocity and stride length, increased stance time and stride width during step turns compared to those of straight walking. However, the current evidence is limited.

There had been no previous study to compare MoS of healthy young adults during straight line walking, step turn and spin turn and this was the first aim of this study. In addition, spatiotemporal parameters were also comprehensively compared in three conditions. We hypothesized that both MoS and stride width of step turn would be greater while those of spin turn smaller than straight walking [8, 13]. We also hypothesized that for both turn conditions, walking speed, step length and stride length would be smaller whereas temporal parameters larger than for straight walking.

## Methods

### Participants

Fifteen healthy young adults [six males, nine females; age, 23.6 (3.1) years; mass, 55.6 (12.2) kg; height, 1.686 (0.105) m; body mass index, 19.4 (2.3) kg·m^-2^; leg length (measured as the distance between the greater trochanter and the floor), 0.850 (0.064) m; mean (standard deviation)] from Tianjin University were included in this analysis. All participants are right-footed determined by kicking a ball [26]. All participants had no history of any neuromusculoskeletal disorders and did not undergo any strenuous physical exercise during 3 days before the experiment. The study was approved by the local ethical committee and all participants gave written informed consent prior to their participation.

### Experimental procedures and data collection

**Fig. 1:**
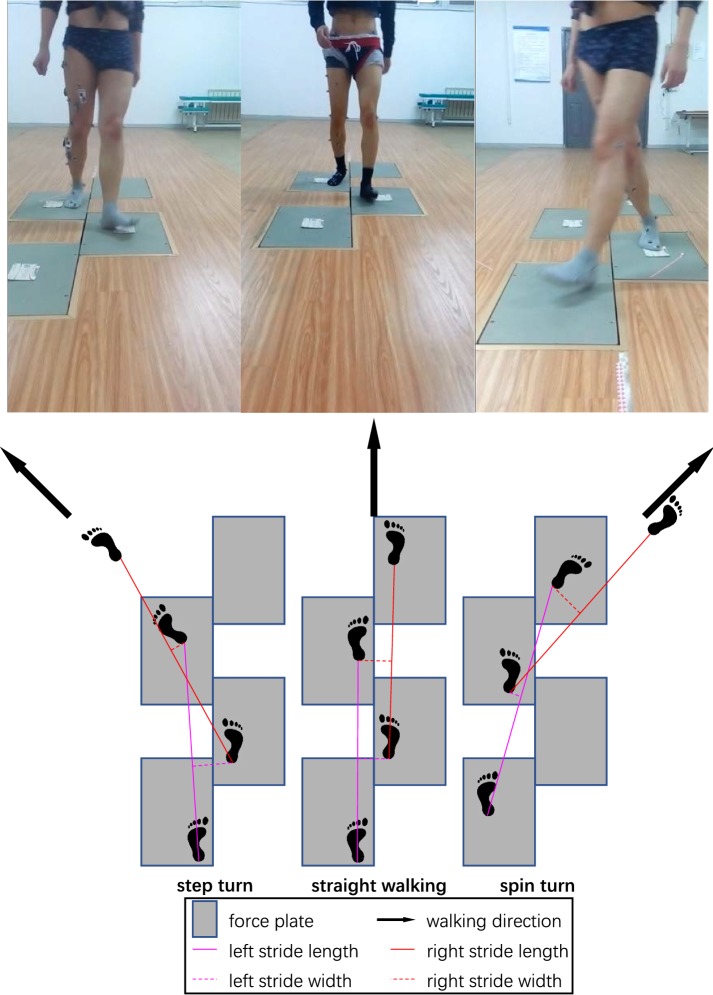
Three barefoot walking tasks: step turn, straight walking and spin turn. Gray rectangles illustrate the relative position of four force plates (40 × 60 cm) embedded in the middle of the walkway (2.5 × 10 m). Arrows in the floor surface pointed to the walking direction

Participants were instructed to perform practice sessions and then complete three barefoot walking tasks at the self-selected preferred walking speed: straight walking, 45° step turn to the left and 45° spin turn to the right (Right lower limb, the dominant leg, was the turning limb in both turn conditions), see Fig. 1 and the additional videos (Additional file 1). A 45° turning angle was chosen due to moderate intensity [27] and easy implementation. Each task consisted of four trials. Marker position data were collected using a 10-camera VICON motion capture system (Vicon Motion Systems Ltd, Oxford, UK) at 100 Hz. Kinetic data were obtained from four AMTI force plates (Advanced Mechanical Technology, Inc., Watertown, MA, USA) sampling at 1000 Hz. Finally, C3D files were imported into MATLAB (R2012b, The MathWorks Inc., Natick, MA, USA) by using the powerful open-source Biomechanical ToolKit [28]. Data processing was performed by a custom program written in MATLAB.

### Data processing

The present study only analyzed the trajectory data of 10 reflective markers placed bilaterally over anatomical landmarks on anterior and posterior superior iliac spine (ASIS and PSIS), lateral malleolus, heel and tip of the toe. Raw marker position data were low-pass filtered using a fourth-order zero-lag Butterworth filter with a cut-off frequency of 4 Hz. All gait data were analyzed in the duration from the first heel strike (HS1) to the third toe off (TO3) (Fig. 1). Heel strike (HS) and toe off (TO) events were identified using force plate data, while the HS4 events of turning conditions were detected as the instant where the heel marker reached local minimum in the vertical direction.

Based on assumptions of inverted pendulum model, extrapolated center of mass (XCoM) and MoS were computed as follows [18]:

1 $$ \begin{aligned} &{\mathbf{XCoM}} = {\mathbf{CoM + }}\frac{{{\mathbf{v}}_{\text{CoM}} }}{{\omega_{0} }} \hfill \\ &\omega_{0} = \sqrt {g/l} \hfill \\ &{\mathbf{MoS}} = {\mathbf{BoS}} - {\mathbf{XCoM}} \hfill \\ \end{aligned} $$

where **CoM** and **v**_CoM_ are the instantaneous position and velocity of whole-body CoM, *ω*_0_ is the (angular) eigenfrequency of the pendulum, *g* is the earth gravitational acceleration (*g* = 9.801 m/s^2^ in Tianjin, China), *l* is the equivalent length of pendulum defined as the distance between CoM and the lateral malleolus, and **BoS** is the anterior, posterior, left or right boundary of BoS (during double support phase) or virtual BoS (during swing phase) [10]. Anterior MoS (MoS_A_) was computed as the distance from XCoM to the line through left and right toe markers, while posterior MoS (MoS_P_) the distance from XCoM to the line through bilateral heel markers. Left and right MoS (MoS_L_ and MoS_R_) were defined as the distance from left and right lateral malleolus to the line between CoM and XCoM. Positive and negative MoS values suggested stable and unstable states (XCoM inside and outside BoS), respectively. We computed MoS at every time point, mean MoS over whole stance phase (including double support period) and MoS at key events (heel strike and mid stance [29]). Considering the convenience (a few markers required) and high validity, we estimated whole-body CoM position using center of pelvis model, as the centroid of the triangle formed by two ASIS markers and the midpoint between two PSIS markers [30]. Instantaneous velocity of CoM (**v**_CoM_) was computed using a first order central difference method. Walking speed was computed as average speed of CoM, viz. the distance travelled divided by the duration of the interval.

Turning gait can be commonly separated into approach, turn, and depart phases [8, 25, 31]. In our work, we defined each phase as stance phase (i.e. approach: HS1 ~ TO1; turn: HS2 ~ TO2; depart: HS3 ~ TO3), excluding swing phase, to compare between matching steps of different walking tasks and between different steps of the same task.

Walking direction was defined as the angle between the direction of **v**_CoM_ and the *x*-axis of the laboratory coordinate system [32] then averaged over every stance phase. The positive values indicated left turns whereas negative values right turns. Spatiotemporal parameters were computed according to standard methods in [33] and non-dimensionally normalized based on [34]. Periods from the HS1 to the HS3 then to the TO3 were normalized to 101 points (0–100) and 63 points (100–162) [8].

### Statistical analysis

A one-way repeated measures analysis of variance was conducted to test for significant differences between matching steps of different walking tasks and between different steps of the same task. Post hoc analyses using the Bonferroni adjustment were carried out to detect the significance of pairwise comparisons. When to determine the difference between two steps of the same task, a paired-samples t-test was used. Variability of spatiotemporal parameters was estimated using 95% confidence interval (CI). These aforementioned statistical analyses were performed using SPSS (version 20.0, Chicago, IL, USA). Variability of gait variables varying throughout gait cycle was estimated using 95% bootstrap confidence band (CB) [8, 35] in MATLAB. All tests were applied at the α = 0.05 level.

## Results

### Walking direction and speed

**Fig. 2:**
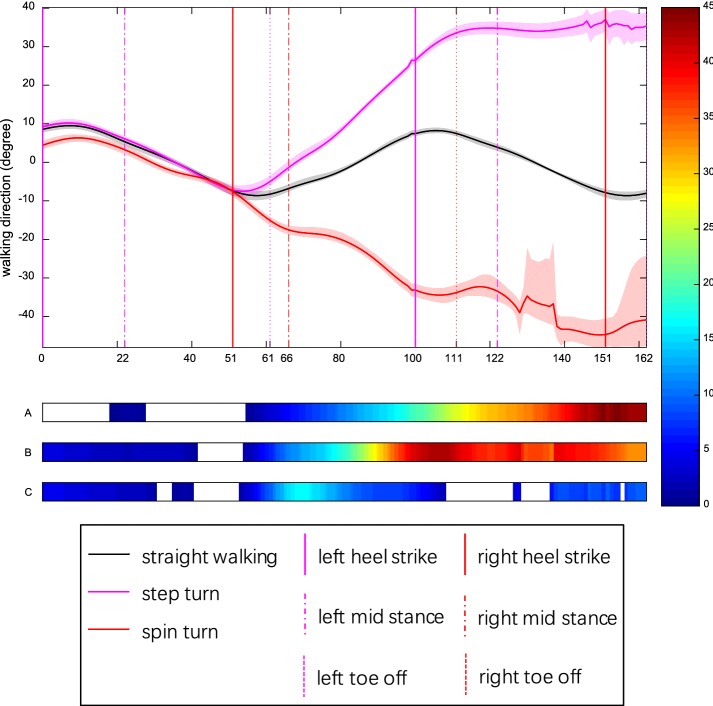
Walking direction for healthy young adults during straight walking, step turn and spin turn. Curves and shadows represent mean and 95% bootstrap CBs. The positive values indicate left turns while negative values right turns. Horizontal colorbars display areas where significant differences (*p* < 0.05) exist (A: step vs straight; B: spin vs straight; C: absolute value of walking direction in step vs spin). Meanwhile, intensity of colorbar indicates difference size from small (blue) to large (red). Color vertical lines show key events: heel strike (HS), toe off (TO), mid stance (MS). Results of time normalization are: HS1—0, MS1—22%, HS2—51%, TO1—61%, MS2—66%, HS3—100%, TO2—111%, MS3—122%, HS4—151%, TO3—162%

The walking direction in straight walking appeared a cosine curve with an amplitude of 10° (Fig. 2). The mean walking direction over every stance phase was closed to zero in three steps of straight walking and approach phase of turns (Additional file 2). The signed walking direction was compared between turns and straight walking (Fig. 2A, B; Additional file 2). The absolute walking direction, i.e. the turning angle magnitude, was compared between step and spin turn, and showed smaller in turn (*p* < 0.0005) and depart (*p* = 0.045) phases of step turn than matching phases of spin turn (Additional file 2†; Fig. 2C). Walking speed was significantly lower in approach and turn phases of step turn compared to counterparts of straight (both *p* ≤ 0.026) and spin (both *p* < 0.0005). There was no significant difference among the third steps of straight walking and turns.

### MoS_A_ and MoS_P_

**Fig. 3:**
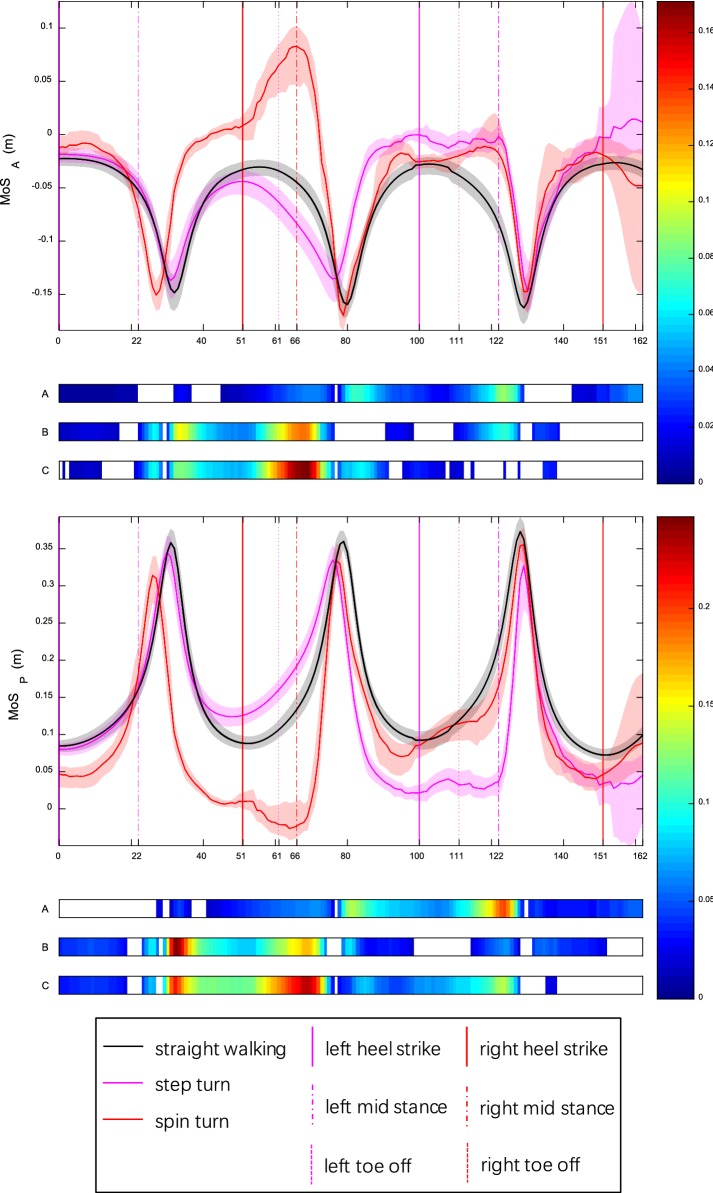
MoS_A_ and MoS_P_ for healthy young adults during straight walking, step turn and spin turn. Curves and shadows represent mean and 95% bootstrap CBs. Horizontal colorbars display areas where significant differences (*p* < 0.05) exist (A: step vs straight; B: spin vs straight; C: step vs spin). Meanwhile, intensity of colorbar indicates difference size from small (blue) to large (red). Color vertical lines show key events: heel strike (HS), toe off (TO), mid stance (MS). Results of time normalization are: HS1—0, MS1—22%, HS2—51%, TO1—61%, MS2—66%, HS3—100%, TO2—111%, MS3—122%, HS4—151%, TO3—162%. For step turn, both MoS_A_ and MoS_P_ were almost consistent with straight walking in the first step. Then MoS_A_ decreased and MoS_P_ increased slightly in the first half of the second step. Finally, MoS_A_ increased and MoS_P_ decreased noticeably. Minimum MoS_P_ appeared in initial and terminal double support phases of the third step. For spin turn, MoS_A_ was greater and MoS_P_ was smaller than straight walking in all three steps. Before the middle of turn phase, MoS_A_ was greater and MoS_P_ was smaller than step turn. Then to the middle of depart, MoS_A_ was smaller and MoS_P_ was greater. Finally, both MoS_A_ and MoS_P_ were almost consistent with step turn. Minimum MoS_P_ occurred at MS2 in turn phase

The curves MoS_A_ and MoS_P_ had opposite shape: an inverted peak vs a peak in each step (Fig. 3). In most conditions, MoS_A_ was negative while MoS_P_ positive. The minimum MoS_A_ occurred at the mid-point of each step whereas the minimum MoS_P_ about heel strike. MoS_P_ of spin turn was smaller than straight walking and step turn in the first and second steps while MoS_P_ of step turn was the smallest of three conditions in the third step. For step turn, mean MoS_P_ over depart was less than turn and approach (both *p* < 0.0005) (Additional file 2). For spin turn, mean MoS_P_ over depart was more than turn and approach (both *p* < 0.0005).

### MoS_L_ and MoS_R_

**Fig. 4:**
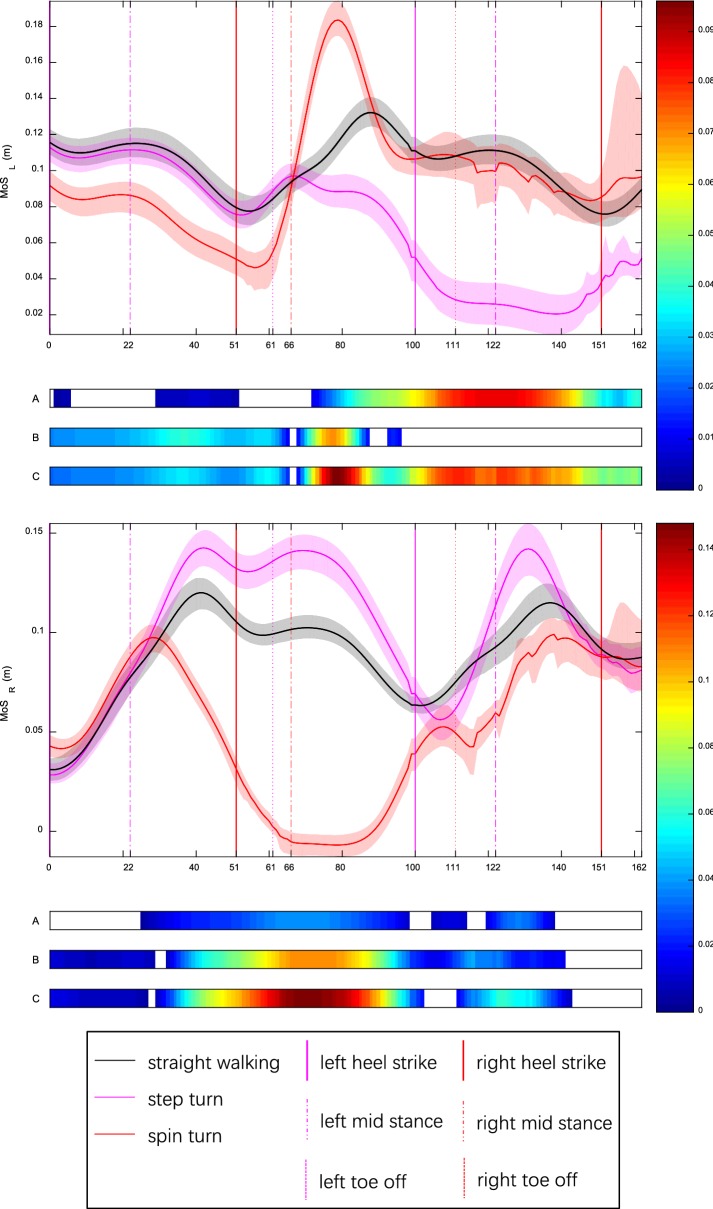
MoS_L_ and MoS_R_ for healthy young adults during straight walking, step turn and spin turn. Curves and shadows represent mean and 95% bootstrap CBs. Horizontal colorbars display areas where significant differences (*p* < 0.05) exist (A: step vs straight; B: spin vs straight; C: step vs spin). Meanwhile, intensity of colorbar indicates difference size from small (blue) to large (red). Color vertical lines show key events: heel strike (HS), toe off (TO), mid stance (MS). Results of time normalization are: HS1—0, MS1—22%, HS2—51%, TO1—61%, MS2—66%, HS3—100%, TO2—111%, MS3—122%, HS4—151%, TO3—162%. For step turn, MoS_L_ was in line with straight walking before MS2 then fell to bottom in depart. MoS_R_ tracked straight walking before MS1 then increased much in turn phase and finally fluctuated around straight walking. For spin turn, MoS_L_ was smaller than straight walking and step turn before MS2 then jumped to a peak in late turn phase and finally varied in concert with straight walking during depart. MoS_R_ was slightly larger than straight walking and step turn before MS1, then plunged to negative bottom in turn phase and finally showed an upturn less than straight walking and step turn in depart

**Fig. 5:**
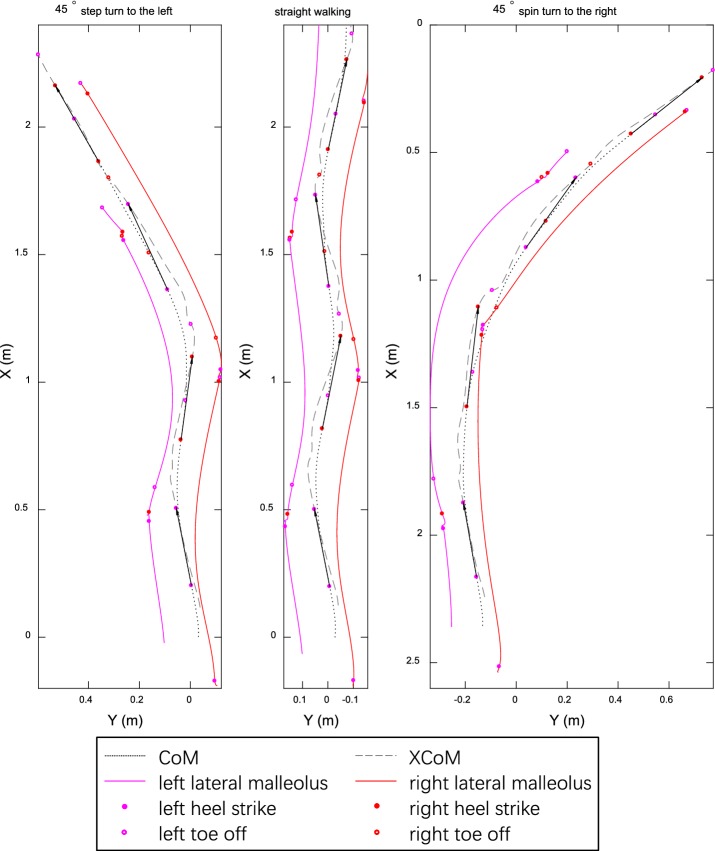
Representative examples of CoM, XCoM and lateral malleolus trajectories for a subject. Black arrows point from CoM to XCoM at HS. Color marker specifiers show key events. The CoM for spin turn was found to be outside the right lateral malleolus during single support (TO1 ~ HS3) of turn phase but XCoM not

For MoS_L_, spin turn was smaller than other conditions in the first step whereas step turn was the smallest in the second and third step. MoS_R_ of spin turn was the smallest in all three steps. For step turn, mean MoS_L_ over depart was less than turn phase (*p* < 0.0005) which in turn was less than approach (*p* < 0.0005) (Additional file 2). Minimum MoS_R_ appeared at right pre-swing (left loading response) (Fig. 4). For spin turn, mean MoS_R_ over turn phase was less than approach (*p* < 0.0005) which in turn was less than depart (*p* = 0.003). Minimum MoS_L_ existed at left pre-swing (right loading response). Example trajectories of CoM, XCoM and lateral malleoli are shown in Fig. 5.

### Spatiotemporal parameters

Compared to straight gait, stride width was wider in turn phase (*p* < 0.0005) and much narrower (*p* < 0.0005) in depart of step turn (Additional file 2). For spin turn, on the other hand, stride width was far narrower in turn (*p* < 0.0005) and little wider in depart (*p* = 1.000). Negative values indicated that the stance foot landed laterally to the swing foot stride vector. Step and stride length were longer in spin condition than in step and straight (all *p* ≤ 0.002). The stance time, single support time, and cycle time were all significantly longer in turns than straight (all *p* ≤ 0.016).

## Discussion

The present study investigated the dynamic stability, as measured by MoS, and spatiotemporal parameters of healthy young adults during straight line walking, step turn and spin turn. This is crucial in rehabilitation evaluation and training for people with risk of falling.

Accurate estimation of the CoM position and velocity is a major factor enabling the computation of MoS. There exist several types of methods for estimation of CoM movement that differ in underlying assumptions and limitations [36]. Generally speaking, the segment-based approach and ground reaction force-based approach [37, 38] have similar performance [36, 39] and are considered as gold standards [38, 40]. Havens et al. [40] computed anteroposterior and mediolateral MoS through four simplified CoM models during straight walking and turning tasks, and assessed their biases and sources of bias via comparing to the gold standard. Their results indicated that bias was larger during turning tasks than straight walking and also showed that bias was smallest when using lower limb plus trunk segment model and center of pelvis. Considering the convenience (a few markers required) and high validity, we estimated the CoM position using center of pelvis model then computed the MoS of every data point in anterior, posterior, left and right directions. The variability was described using 95% bootstrap CB, a continuous data analysis procedure, rather than point-by-point Gaussian-based CI resulting in increased type I error rates [8, 35].

Fifteen subjects were instructed to perform straight walking and turns at comfortable speed. Results showed that the real turn angle in spin turn was slightly greater than in step turn. Broadly speaking, step turn was a bit slower whereas spin turn faster than straight walking. These are inconsistent with what we hypothesized and previous studies [8, 13] and reveal that spin turn might be a high efficiency strategy to change the direction of progression largely and quickly in healthy young adults [41].

MoS_A_ was an inverted peak in each step and the minimum value occurred at the mid-point of each stance phase, which is also the middle of swing phase on contralateral side. It suggests that people are most likely to fall forward at the middle of swing phase in straight walking and turns. This result is in agreement with a recent study [42] which indicated that the greatest risk of trip-related falls occurred at the middle of swing phase. In contrast, MoS_P_ presented a peak in each step and the minimum value appeared at heel strike. It indicates that people are most likely to fall backward at heel strike. This result is in line with [43] which reported that the greatest risk of slip occurred at the time shortly after heel strike and resulted in backward fall in normal level walking. The negative MoS_A_ leads to step forward because the maximum MoS_A_ will occur at heel strike. In other words, walking cannot be stopped during the step period, especially at the middle of swing phase where the minimum MoS_A_ occurred, which indicates that the negative MoS_A_ maybe characterize the difficulty of stopping within one step [15]. The negative value also reveals that people prefer falling forward instead of backward when balance is threatened [44]. This may be due to body inertia, or probably because people could better reduce the risk of head and spine impacting on lower surfaces to protect central nervous system with the help of vision and upper limbs when falling forward.

For straight walking, lateral MoS grew down during stance phase (the first and third step on left side and the second step on right side, Fig. 4) in which both CoM and XCoM were close to stance limb (Fig. 5). Conversely, lateral MoS went up during swing phase (the first and third step on the right and the second step on the left). Minimum lateral MoS was located at contralateral heel strike during straight walking, turn stance phase of spin turn and depart stance phase of step turn. This suggests that people are most likely to fall toward the support side at contralateral heel strike during straight walking, turn phase of spin turn and depart phase of step turn.

In general, there were almost the same size relationships of MoS, represented by four common characterizations, mean and minimum MoS and MoS at heel strike and mid stance, between three conditions (Additional file 2). We also found that the mid-point of stance phase or the middle of swing phase was vital for MoS_A_ and MoS_P_.

For stride width, step turn phase was a good bit wider while depart a lot narrower. In contrast, spin turn phase was a great deal narrower whereas depart somewhat wider. This is in keeping with [8, 12]. We believe that this is the two natural cooperating strategies for the bilateral lower limbs to complete the turn within only three steps. For step length and stride length, spin turn was the longest. This implies that spin turn takes a long path to succeed in altering direction. The stance time, single support time, and cycle time increased in turns than straight. Precisely speaking, it is mainly because that the single support time or swing time gets longer in turns than straight walking.

Two limitations may have affected this current study. First, the embedded force plates may affect the subjects’ natural walking pattern [45]. Future studies should consider choosing portable force plates and blinding their locations to the participants, or using cutting-edge technology such as [45, 46]. Second, the estimation of CoM position using center of pelvis model rather than gold standard might be not precise enough. This simplified CoM model would result in similar overestimation of MoS_A_ whereas similar underestimation of MoS_P_ between straight walking, step turn and spin turn, but underestimation of the lateral MoS during stance phase only in spin turn [40]. Therefore, future research should include consideration of more accurate estimation methods.

## Conclusions

In conclusion, MoS_A_ reaches a minimum at the middle of swing phase in straight walking and turns. The backward instability threatens the first half of spin turn and the last half of step turn. Both backward and contralateral MoS appear a minimum at heel strike. Great lateral instability and negative stride width occur in the depart phase of step and the turn phase of spin. Spin turn takes a long step and stride length. Both turns undergo long swing phases.

For people with poor balance function, turning gait might expose potential problems which are undetectable in straight walking. This work could provide a valuable reference for rehabilitation evaluation and training for patients with cerebral palsy [9], post-stroke hemiparesis [46, 47], Parkinson’s disease [12, 23], or multiple sclerosis [29]. For example, they could be trained from straight walking to step turn and spin turn, for gradual adaptation of gait stability and spatiotemporal parameters. Additionally, they should be instructed to pay attention to posterior and contralateral stability at heel strike, such as avoid slipping, and anterior stability at the middle of swing phase, such as avoid tripping.

Future studies overcoming the limitations are required to further understand preference strategy, kinematics, kinetics and bilateral asymmetry during planned and unplanned turning at different angles and velocities in older adults and people with cerebral palsy, post-stroke hemiparesis, Parkinson’s disease, or multiple sclerosis.

## Additional files

**Additional file 1.** Example videos of three barefoot walking tasks: step turn, straight walking and spin turn. Participants were instructed to perform practice sessions and then complete three barefoot walking tasks (wearing socks if cold) at the self-selected preferred walking speed: straight walking, 45° step turn to the left and 45° spin turn to the right (Right lower limb is the turning limb in both turn conditions). Four force plates were used in straight walking while three in turns.

**Additional file 2.** Spatiotemporal parameters, MoS_A_, MoS_P_, MoS_L_ and MoS_R_ for healthy young adults (*n* = 15). Mean (95% confidence interval). Statistically significant difference (*p* < 0.05) from the first phase of same condition (a), the second phase of same condition (t), straight walking (*), between turn conditions (†). ‡: Statistically significant difference (*p* < 0.05) between the absolute mean value of walking direction in spin turn and that in step turn.

## Abbreviations

BoS: base of support
CoM: center of mass
**v**_CoM_: velocity of center of mass
XCoM: extrapolated center of mass
MoS: margin of stability
MoS_A_: anterior margin of stability
MoS_P_: posterior margin of stability
MoS_L_: left margin of stability
MoS_R_: right margin of stability
ASIS: anterior superior iliac spine
PSIS: posterior superior iliac spine
HS: heel strike
HS1: the first heel strike
HS2: the second heel strike
HS3: the third heel strike
HS4: the fourth heel strike
TO: toe off
TO1: the first toe off
TO2: the second toe off
TO3: the third toe off
MS: mid stance
MS1: the first mid stance
MS2: the second mid stance
MS3: the third mid stance
CI: confidence interval
CB: confidence band
