# Supplementary figures and images for: Dynamic stability and spatiotemporal parameters during turning in healthy young adults
Source: Biomed Eng Online. 2018 Sep 21;17:127. doi: 10.1186/s12938-018-0558-5 (PMC6151057; doi:10.1186/s12938-018-0558-5)

## Slide 1
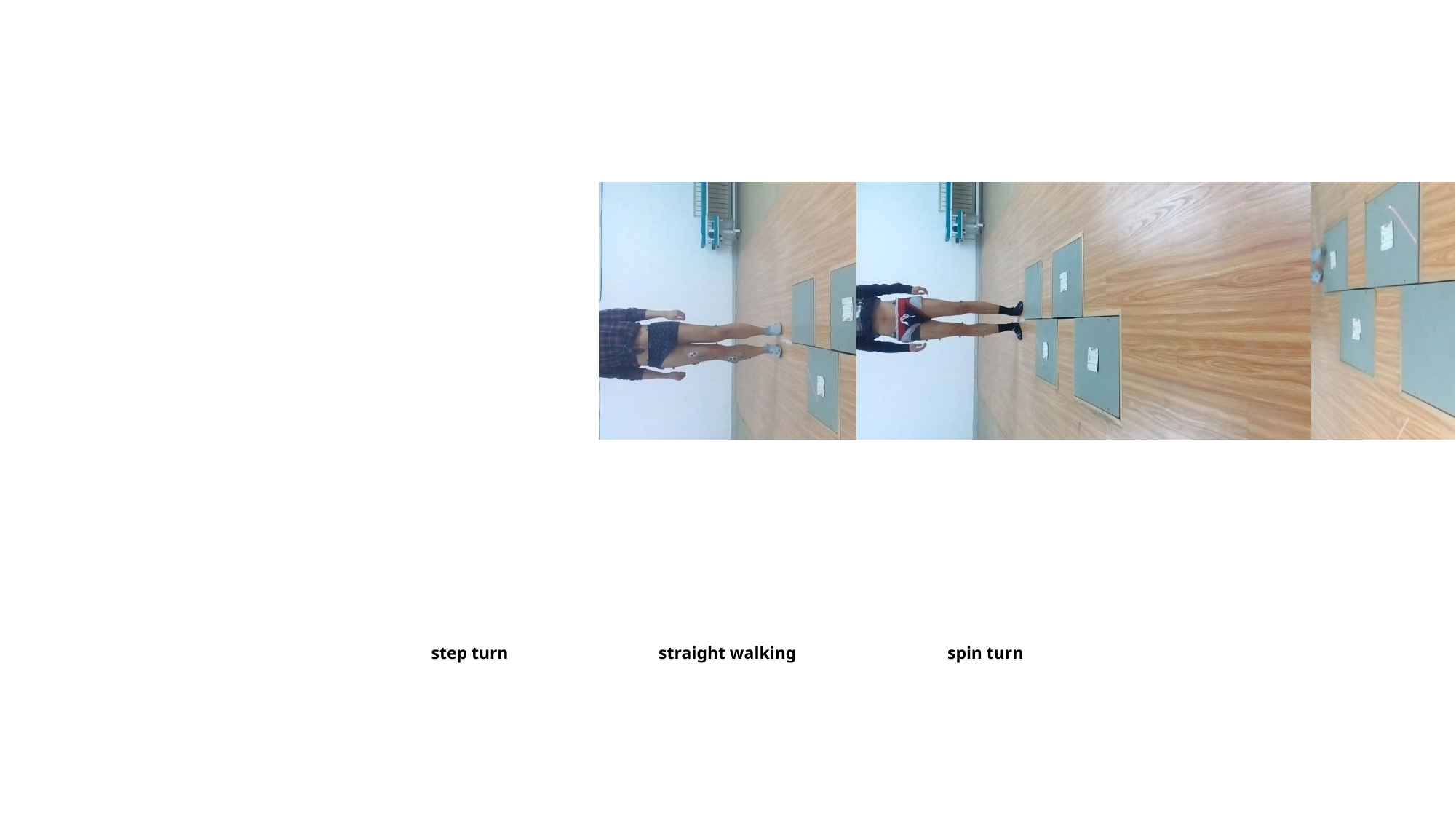

step turn
straight walking
spin turn

Supplement: Supplementary file 1 — Additional file 1. Example videos of three barefoot walking tasks: step turn, straight walking and spin turn. Participants were instructed to perform practice sessions and then complete three barefoot walking tasks (wearing socks if cold) at the self-selected preferred walking speed: straight walking, 45° step turn to the left and 45° spin turn to the right (Right lower limb is the turning limb in both turn conditions). Four force plates were used in straight walking while three in turns. [file 12938_2018_558_MOESM1_ESM.pptx]
